# Complement Gene Variants Define the Conformational Dynamics of C3 Convertase

**DOI:** 10.1002/eji.70242

**Published:** 2026-07-22

**Authors:** Héctor Martín Merinero, Seth J. Welsh, Zhen Xu, Richard J. H. Smith, Yuzhou Zhang

**Affiliations:** ^1^ Molecular Otolaryngology and Renal Research Laboratories University of Iowa Iowa City Iowa USA; ^2^ Protein Structure, Analysis, & Design Core University of Iowa Iowa City Iowa USA

## Abstract

Current understanding of the structure, function, and molecular dynamics of the alternative pathway C3‐convertase has been shaped by the clinical impact of genetic variants in complement genes. Characterizing the functional impact of different mutations not only provides patient‐specific insights into the pathogenic mechanisms underlying a number of complement‐mediated diseases but, at a broader level, allows us to dissect the molecular underpinnings of protein‐protein interactions that drive complement amplification and its control. In this review, we contextualize over 100 variants in *C3*, *CFB*, *CFD*, *CFH*, and *CFI* to illustrate how this collective knowledge informs known mechanisms of complement biology and illuminates gaps that remain to be defined.

## Graphical abstract

Multidisciplinary data have significantly advanced our understanding of C3bBb molecular dynamics. This review specifically contextualizes the functional impact of disease‐associated genetic variants, demonstrating that clinical genetics not only supports the current C3bBb model but also provides novel insights into previously undefined molecular mechanisms.

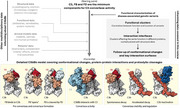

## 1 Introduction

The complement system is the cornerstone of innate immunity. Activity is initiated by three distinct pathways—the classical (CP), lectin (LP) and alternative (AP)—with subsequent activation following a well‐defined enzymatic cascade: an initial trigger activates a zymogen, which activates another zymogen, in a continual process of proteolytic cleavage that ultimately leads to the generation of different molecular complexes known as C3‐convertases: C4b2b for the CP/LP, and C3bBb for the AP [1]. Of these two C3 convertases, the latter is foundational to complement biology, driving amplification of initial complement signals through a positive feedback/amplification loop. The product of C3bBb serine protease activity is C3b, which is the platform for the formation of additional C3bBb [2]. So robust is this amplification process that 80% of ALL complement activity has been attributed to the AP C3‐convertase independent of the initiating trigger [3].

**FIGURE 1:**
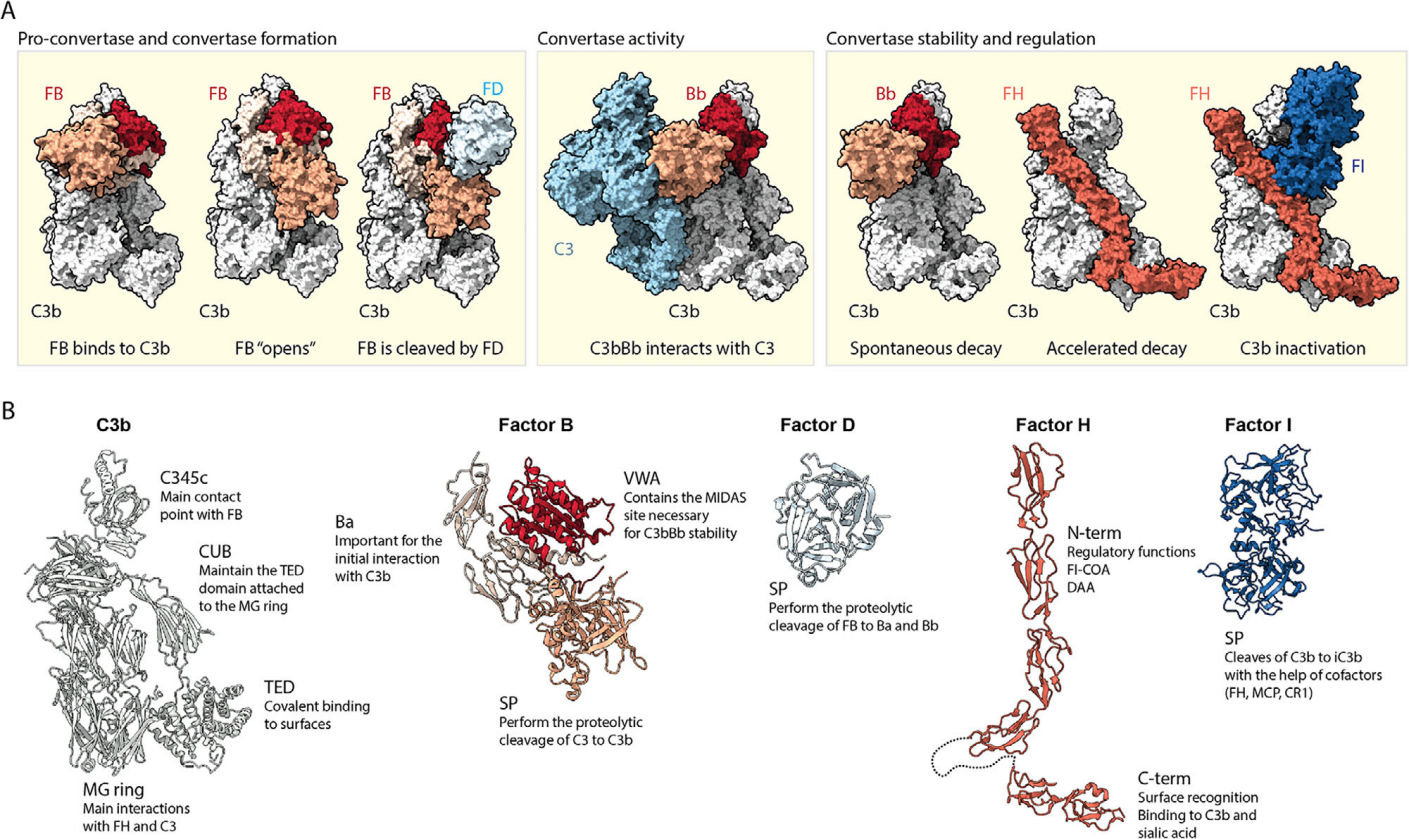
C3bBb model and participating proteins. (A) Surface representation of C3bBb model: formation, activity, and regulation of C3bBb. From left to right, the following discrete steps are depicted: FB binds to C3b (FB_Close_ from PDB 3HRZ represented on C3b from PDB 2I07), FB changes conformationally from “closed” to “open” (PDB 2XWJ), FD binds and cleaves FB (PDB 2XWB), C3bBb interacts and cleaves C3 substrate (PDB 9U62), C3bBb dissociates spontaneously (C3bBb from PDB 9U62), FH binds to C3b to accelerate the decay of the C3bBb (PDB 5O35), and FI inactivates C3b to iC3b with FH as cofactor (PDB 5O32). (B) Ribbon representation of the proteins participating in C3bBb formation, activity, and regulation, highlighting their domains and associated functions. From left to right: C3b (PDB 2I07), FB (extracted from PDB 3HRZ), FD (extracted from PDB 2XWB), FH (extracted from PDB 5O35), FI (extracted from PDB 5O32). C3b is represented in white (#f7f7f7), FB is domain‐colored (Ba is light orange #fddbc7; vWA is dark red #b2182b; SP is orange #f4a582), FD is represented in pale blue (#d1e5f0), C3 is represented in light blue (#92c5de), FH is represented in dark orange (#d6604d) and FI is represented in dark blue (#2166ac).

The origins of our understanding of C3bBb were grounded in the seminal work of Fearon, identifying C3, FB, and FD as the minimum protein components required to generate C3‐convertase activity in the presence of magnesium [4]. Today, collective knowledge has refined this insight into a complex three‐step model of convertase formation, activity, and dissociation that includes several intermediate molecular states (Figure 1A). The current model of C3bBb dynamics is supported by multiple lines of evidence generated from structural (crystallography, electron microscopy, cryogenic electron microscopy), functional (antibodies, nanobodies, protein mutants), mammalian (rodents, canines, nonhuman primates), and patient data (biomarkers, complement inhibitor effects, clinical trial outcomes) that shape our understanding of C3bBb dynamics. In this review, we focus on how the functional characterization of disease‐associated complement gene variants informs our understanding of C3bBb and illuminates knowledge gaps yet to be defined.

## 2 Convertase Model and Participating Proteins

Briefly, the C3bBb model proceeds as follows: FB binds to C3b (the “closed” proconvertase: C3Bb_Closed_) and undergoes a conformational change (“open” proconvertase: C3Bb_Open_) that allows FD to bind and cleave FB, releasing Ba and reorienting Bb to form the functional C3bBb convertase [5, 6, 7]. The C3bBb convertase can now bind and cleave nearby C3 or dissociate spontaneously—90 s half‐life [8]—or in an accelerated manner via regulators of complement activity (RCA). FI disrupts this process by inactivating C3b to iC3b together with other RCAs that act as cofactors, preventing the formation of new convertases (Figure 1A).

To contextualize the functional impact of the variants characterized in the literature, it is necessary to understand the role of the various domains of the different proteins involved in the formation, activity, and regulation of C3bBb: C3/C3b, FB, FD, and RCA (Figure 1B).

C3 belongs to the macroglobulin family and is therefore composed of eight macroglobulin (MG) domains arranged in a ring‐shaped structure [9]. The anaphylatoxin domain of C3, C3a is released via proteolytic cleavage when C3 is activated to C3b. This conformational change to C3b exposes a thioester bond in the thioester‐containing domain (TED), which covalently binds to nearby surfaces. The TED is linked to the rest of the molecule via the CUB domain. The remaining terminal carboxyl residues constitute the C345c domain, which establishes a magnesium‐dependent interaction with FB. The MG‐ring in C3b establishes the primary interactions with FB to assemble the convertase, with the C3 substrate during the activation process, and with FH or other RCA during the regulation of C3bBb and C3b.

Factor B (FB) is the catalytic component of the C3bBb complex. It consists of three short consensus repeats (SCR) domains (globular domains of approximately 60 amino acids that serve as structural subunits within complement proteins), grouped in the Ba fragment, that participate in the initial interaction with C3b; a von Willebrand type A (vWA) domain that mediates the magnesium‐dependent interaction with the C345c domain of C3b via the metal‐ion‐dependent adhesion site (MIDAS); and a serine protease (SP) domain responsible for the catalytic activity of the convertase, cleaving C3. The vWA and SP domains constitute the Bb fragment of FB.

Factor D (FD) is the serine protease responsible for cleaving FB into Ba and Bb, thereby mediating the transition from proconvertase (C3bB) to convertase (C3bBb). Spontaneous activation of FB in the fluid phase is prevented by the necessary conformational change that FB undergoes only upon binding to C3b. FD consists solely of an SP domain.

Factor H (FH) is the primary regulator of C3bBb and the most studied RCA in the context of disease pathogenesis. FH possesses convertase decay‐accelerating activity (DAA), a function shared by two other RCA proteins: decay‐accelerating factor (DAF) and complement receptor 1 (CR1) [10]. FH is a soluble glycoprotein consisting of 20 SCR domains capable of recognizing self‐surfaces, which allows it to regulate complement in the fluid phase and on surfaces. Its regulatory functions are located at the amino‐terminal end (SCRs1–4), while its surface recognition capacity is located at the carboxy‐terminal end (SCRs19–20), which binds to C3b and self‐surface ligands such as sialic acids [11, 12]. DAF and CR1 are structurally similar to FH, as they consist of SCRs and possess regulatory domains [13]. However, they lack surface recognition domains and are not secreted but rather exist solely as membrane RCAs.

Factor I (FI) is the serine protease responsible for inactivating C3b into iC3b, a process that requires the participation of a cofactor such as FH, membrane cofactor protein (MCP), or CR1. FI binds to the C345c domain of C3b via its FI‐membrane (FIM) domain, and to the C3b CUB domain and to the cofactors via its SP domain. FI also possesses a cysteine‐rich scavenger receptor domain (SRCR) and class A1 and A2 low‐density lipoprotein receptors (LDLr) [14]. The SP domain of FI is responsible for the cleavage of C3b into iC3b and C3f at the CUB domain (cleavage sites R954‐S955 and R1303‐S1304) [15], and for the cleavage of iC3b into C3c and C3dg, for which only CR1 can act as a cofactor under physiological conditions [16, 17].

The structures depicting each step of the C3bBb model, which will be referred to throughout the review, were generated in ChimeraX using structures available in the Protein Data Bank (PDB). For the structures of C3bB_Open_, C3bB_Open_+FD, C3bBb‐C3, C3bBb, C3b‐FH, and C3b‐FH‐FI, PDB files 2XWJ, 2XWB, 9U62, 5O35, and 5O32 were used with no modifications other than hiding other proteins present in the files when necessary. In the absence of a C3Bb_Close_ structure, the FB_Close_ structure from PDB 3HRZ was aligned to the C3b structure from PDB 2I07 by aligning the MG rings of cobra venom factor (CVF) and C3b, using a similar approach to that employed by Forneris [5]. Due to the high mobility of the C345c domains between structures, the C345c domains were aligned independently. The footprints of the interactions are shown to highlight the relevant interaction surfaces. These have been defined by proximity using the PDBsum software tool from EMBL‐EBI [18].

## 3 Variant Characterization Provides Insights Into C3bBb Structure and Function

Over the past 30 years, numerous variants of the proteins involved in the C3bBb model—whether associated with complement‐mediated diseases or engineered in the laboratory—have been functionally characterized, providing mechanistic insight into both disease pathogenesis and basic complement function.

### 3.1 FB Binding to C3b and Conformational Change of the Pro‐Convertase

**FIGURE 2:**
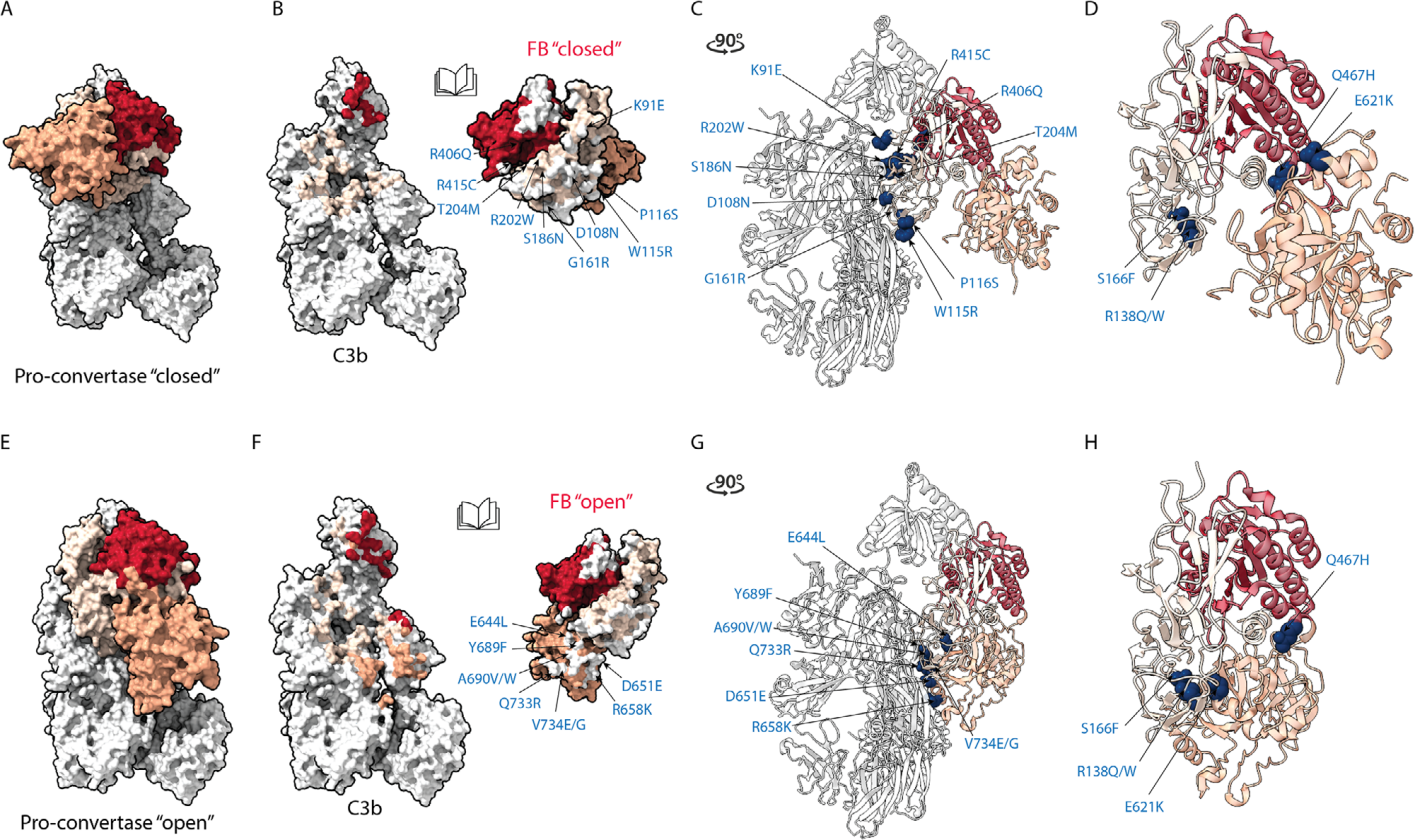
Mutants informing of FB's binding to C3b and conformational change. (A) Surface representation of C3bB_Close_ (FB_Close_ from PDB 3HRZ represented on C3b from PDB 2I07). (B) Open book representation of (A), highlighting the footprint of the interaction between FB_Close_ and C3b. Mutants in the Ba and vWA domains of FB affecting the binding to C3b are indicated. (C) 90° anticlockwise‐rotated ribbon representation of (A). Mutants in the Ba and vWA domains of FB affecting the binding to C3b are represented as deep dark blue (#053061) spheres. (D) Ribbon representation of FB_Close_ (from PDB 3HRZ) in the same orientation as (C). Mutants away from the footprint surface are indicated as deep dark blue (#053061) spheres. (E) Surface representation of C3bB_Open_ (PDB 2XWJ). (F) Open book representation of (D), highlighting the footprint of the interaction between FB_Open_ and C3b. Mutants in the SP domain of FB affecting the binding to C3b are indicated. (G) 90° anticlockwise‐rotated ribbon representation of (D). Mutants in the SP domain of FB affecting the binding to C3b are represented as deep dark blue (#053061) spheres. (H) Ribbon representation of FB_Open_ (from PDB 2XWJ) in the same orientation as (F). Mutants away from the footprint surface are indicated as deep dark blue (#053061) spheres. C3b is represented in white (#f7f7f7), and FB is domain‐colored (Ba is light orange #fddbc7; vWA is dark red #b2182b; SP is orange #f4a582).

The starting point of the C3bBb model is the binding of FB to C3b, and it has been observed that several FB variants directly influence this interaction (Table 1). During this initial interaction, the FB footprint on C3b is mostly covered by its Ba domain (Figure 2A,B). Accordingly, many of the described variants affecting binding are located on the surface of the Ba domain in contact with C3b (Figure 2B,C) [19]. Interestingly, the remaining variants do not provide information about the initial interaction, but rather about the conformational change that FB undergoes when transitioning from C3bB_Close_ to C3bB_Open_. Most variants cluster on the surface of the SP domain and define the footprint of FB on C3b observed in C3bB_Open_ (Figure 2D–F). The remaining nonfootprint variants affect residues R138, S166, and E621, which are physically separated from one another and from C3b in C3bB_Close_, but come into direct contact with each other in C3bB_Open_, likely participating in the maintenance of the “open” conformation (compare Figure 2D with 2H). The residue Q467 is located near the loop connecting the vWA and SP domains (residues 470–478), which undergoes the rotation for the “opening” of FB (Figure 2G,H). Variants in these residues may be affecting these two events, but this requires further demonstration.

**TABLE 1:** Variants informing about FB binding to C3b and pro‐convertase conformational change (*n* = 26).

Gene	cDNA	Exon	Protein	Domain	Informs about	Reference
**FB**	c.94C>T	2	R32W	Ba	Initial binding to C3b (FB_Closed_)	[45]
	c.95G>A	2	R32Q	Ba	Initial binding to C3b (FB_Closed_)	[45]
	c.271A>G	2	K91E	Ba	Initial binding to C3b (FB_Closed_)	[19]
	c.322G>A	3	D108N	Ba	Initial binding to C3b (FB_Closed_)	[19]
	c.343T>C	3	W115R	Ba	Initial binding to C3b (FB_Closed_)	[19]
	c.346C>T	3	P116S	Ba	Initial binding to C3b (FB_Closed_)	[19]
	c.412C>T	3	R138W	Ba	Conformational change (C3bB_Closed_ → C3bBb_Open_)	[19]
	c.413G>A	3	R138Q	Ba	Conformational change (C3bB_Closed_ → C3bBb_Open_)	[19]
	c.481G>A	4	G161R	Ba	Initial binding to C3b (FB_Closed_)	[19]
	c.497C>T	4	S166F	Ba	Conformational change (C3bB_Closed_ → C3bBb_Open_)	[19]
	c.557G>A	4	S186N	Ba	Initial binding to C3b (FB_Closed_)	[19]
	c.604C>T	4	R202W	Ba	Initial binding to C3b (FB_Closed_)	[19]
	c.611C>T	4	T204M	Ba	Initial binding to C3b (FB_Closed_)	[19]
	c.1217G>A	9	R406Q	vWA	Conformational change (C3bB_Closed_ → C3bBb_Open_)	[19]
	c.1243C>T	9	R415C	vWA	Conformational change (C3bB_Closed_ → C3bBb_Open_)	[19]
	c.1401A>T	10	Q467H	vWA‐SP	Conformational change (C3bB_Closed_ → C3bBb_Open_)	[19]
	c.1861G>A	15	E621K	SP	Conformational change (C3bB_Closed_ → C3bBb_Open_)	[19]
	*In‐lab designed*	15	E644L	SP	Conformational change (C3bB_Closed_ → C3bBb_Open_)	[5]
	c.1953T>G	15	D651E	SP	Conformational change (C3bB_Closed_ → C3bBb_Open_)	[19]
	c.1973G>A	16	R658K	SP	Conformational change (C3bB_Closed_ → C3bBb_Open_)	[19]
	*In‐lab designed*	16	Y689F	SP	Conformational change (C3bB_Closed_ → C3bBb_Open_)	[5]
	c.2069C>T	16	A690V	SP	Conformational change (C3bB_Closed_ → C3bBb_Open_)	[19]
	*In‐lab designed*	16	A690W	SP	Conformational change (C3bB_Closed_ → C3bBb_Open_)	[5]
	*In‐lab designed*	18	Q733R	SP	Conformational change (C3bB_Closed_ → C3bBb_Open_)	[5]
	c.2201T>A	18	V734E	SP	Conformational change (C3bB_Closed_ → C3bBb_Open_)	[19]
	*In‐lab designed*	18	V734G	SP	Conformational change (C3bB_Close_ → C3bBb_Open_)	[5]

### 3.2 FB Cleavage by FD

**FIGURE 3:**
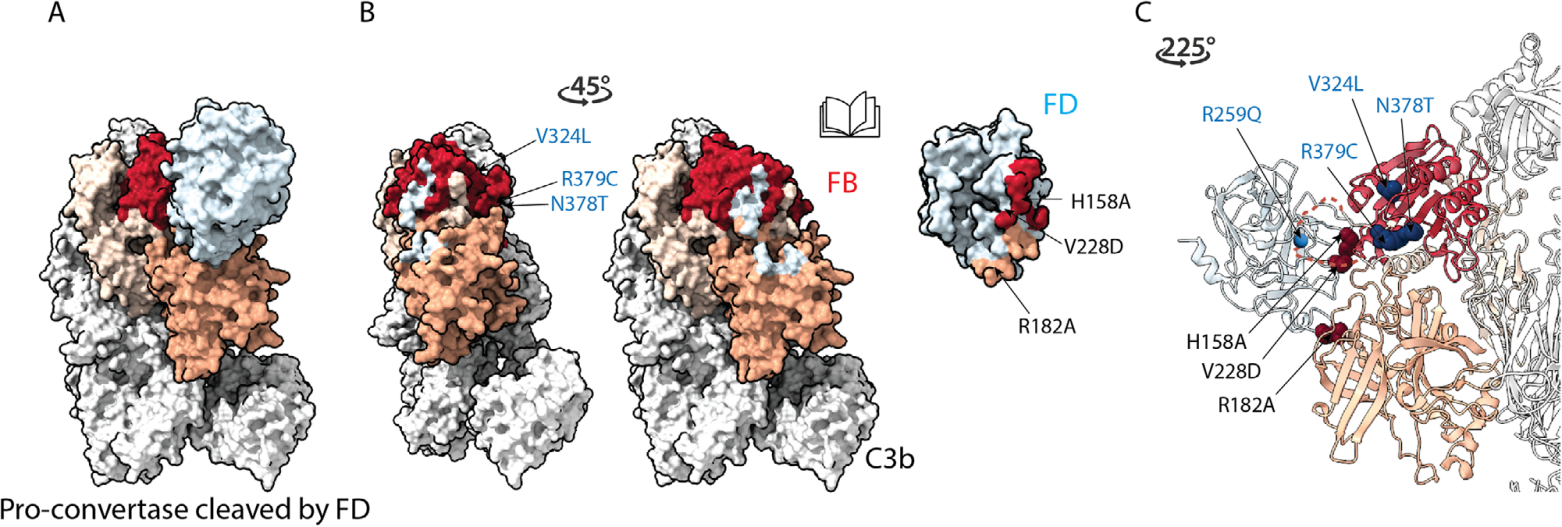
Mutants informing of FD binding to FB and exposition of FB's scissile loop. (A) Surface representation of C3bB_Open_‐FD (PDB 2XWB). (B) Open book representation of (A), highlighting the footprint of the interaction between FD and FB. In‐lab designed FD mutants preventing their activity are indicated. An additional 45° anticlockwise rotated perspective of C3bB_Open_ is represented on the left to enable visualization of the mutants in the vWA domain of FB that prevent the cleavage by FD. (C) 225° anticlockwise‐rotated ribbon representation of (A). In‐lab designed FD mutants and disease‐associated variants in the vWA domain of FB impairing the cleavage by FD are represented as deep dark red (#67001f) and deep dark blue (#053061) spheres, respectively. C3b is represented in white (#f7f7f7), FB is domain‐colored (Ba is light orange #fddbc7; vWA is dark red #b2182b; SP is orange #f4a582), and FD is represented in pale blue (#d1e5f0).

When the FB is “open” in C3bB_Open_, FD can bind and cleave it. A few mutants that alter this process have been described (Table 2). The only known FD variants were designed by Forneris et al. [5] to formally confirm the interaction they observed in the C3bB_Open_‐FD crystal they generated (Figure 3A), and accordingly, alter some of the residues that define the footprint of this interaction (Figure 3B,C). In contrast, the disease‐associated FB variants (R259Q, V324L, N378T and R379C) are located far from the footprint and inform about the exposure of the scissile loop to FD (Figure 3B). Specifically, residue R259 is located within the scissile loop (253‐263) and belongs to the target of FD's catalytic triad (258–259), while the residues V324, N378, and R379 are located in the vicinity of the scissile loop and likely participate in its proper presentation to FD (Figure 3C).

**TABLE 2:** Variants informing of FD binding to FB and exposition of FB's scissile loop (*n* = 6).

Gene	cDNA	Exon	Protein	Domain	Informs about	Reference
**FB**	c.776G>A	6	R259Q	Ba‐vWA	FD target	[19]
	c.970G>T	7	V324L	vWA	Exposition of scissile loop	[19]
	c.1135C>T	8	R379C	vWA	Exposition of scissile loop	[19]
**FD**	In‐lab designed	2	H158A	SP	Binding to C3b	[5]
	In‐lab designed	2	R182A	SP	Binding to C3b	[5]
	In‐lab designed	2	V228D	SP	Binding to C3b	[5]

### 3.3 C3bBb Interaction and Cleavage of C3 Substrate

**FIGURE 4:**
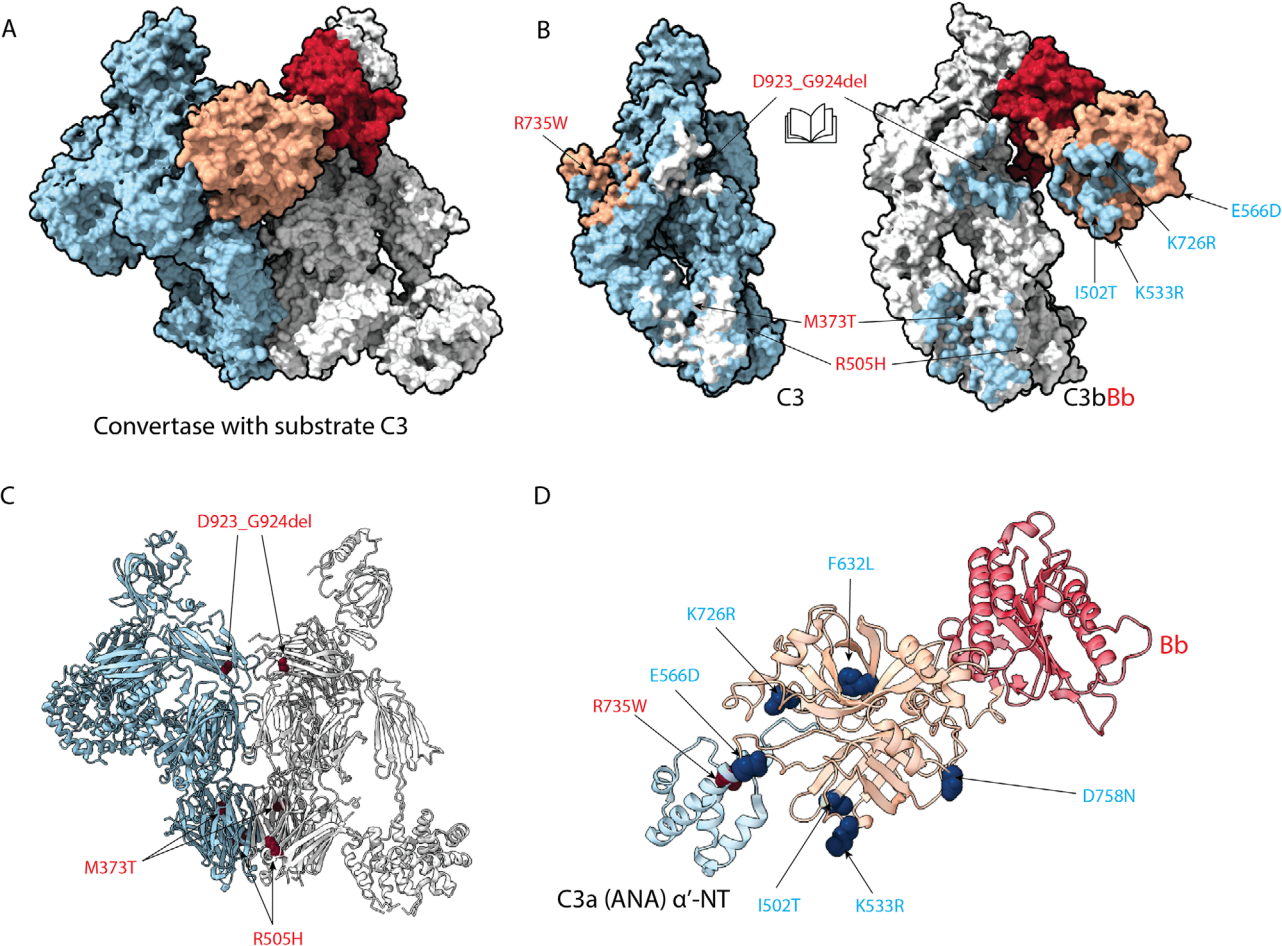
Mutants informing of the interaction C3bBb‐C3. (A) Surface representation of C3bBb‐C3 (PDB 2U62). (B) Open book representation of (A), highlighting the footprint of the interaction between C3bBb and C3 substrate. Mutants impairing C3 activation by C3bBb are indicated, either in the C3 substrate or C3b and Bb components of the C3bBb. Some variants informing of this interaction are not indicated because they are buried or hidden in the perspective represented. (C) Ribbon representation of (A). The Bb component of C3bBb is removed to facilitate the observation of C3–C3b interaction. Mutants are represented as deep dark red (#67001f) spheres. (D) Zoomed ribbon representation of Bb and C3a component of C3 substrate. Mutants in C3 and Bb informing about this interaction are represented as deep dark red (#67001f) and deep dark blue (#053061) spheres, respectively. C3b is represented in white (#f7f7f7), Bb is domain‐colored (vWA is dark red #b2182b; SP is orange #f4a582), and the C3 substrate is represented in light blue (#92c5de).

Once FD cleaves FB, and Ba is released, Bb changes its orientation, forming the transient and labile C3bBb (Figure 1A) [5, 6, 7]. Despite the short‐lived interaction of C3 with C3bBb, some researchers have been able to characterize variants of C3/C3b and FB that provide information about the surfaces involved in the interaction between the convertase and the C3 substrate (Table 3) (Figure 4A). The C3 variants M373T (MG4), R505H (MG5), and del_D923‐G924 (MG7) reduce the rate at which C3bBb activates C3, regardless of whether they are carried by the C3b of the C3bBb or by C3 substrate, revealing important interaction sites between the convertase and its substrate that agree with the footprints on both C3 and C3b (Figure 4B,C) [20, 21, 22]. Similarly, the variants found in FB only affect the C3bBb activity rate, without altering FB binding to C3b or the stability/resistance of C3bBb to RCA regulation [19]. It is worth noting that their location is restricted to the SP domain, around the catalytic triad, and on the binding site with C3a and MG3 or surrounding residues (Figure 4B,D), delineating the interaction surface between the SP domain of FB and the C3 substrate. Also supporting this interaction surface, the C3 R735W variant, which is not functionally characterized but has been consistently associated with complement‐mediated diseases, mirrors the FB variants by being located very close to this interaction surface (Figure 4B,D) [29].

**TABLE 3:** Variants informing of the interaction C3bBb‐C3 (*n* = 10).

Gene	cDNA	Exon	Protein	Domain	Informs about	Reference
**C3**	c.1118T>C	10	M373T	MG4	Interaction C3‐C3b	[22]
	c.1514G>A	13	R505H	MG5	Interaction C3‐C3b	[20]
	c.2203C>T	17	R735W	C3a (ANA)	Binding to FB (SP)	[29]
	c.2767_2774delACGGTG	22	D923_G924del	MG7	Interaction C3‐C3b	[21]
**FB**	c.1505T>C	11	I502T	SP	Binding to C3 substrate	[19]
	c.1598A>G	12	K533R	SP	Binding to C3 substrate	[19]
	c.1698A>C	13	E566A	SP	Binding to C3 substrate	[19]
	c.1896T>G	15	F632L	SP	Binding to C3 substrate	[19]
	c.2177A>G	18	K726R	SP	Binding to C3 substrate	[19]
	c.2272G>A	18	D758N	SP	Binding to C3 substrate	[19]

### 3.4 C3bBb Spontaneous and Accelerated Decay

**FIGURE 5:**
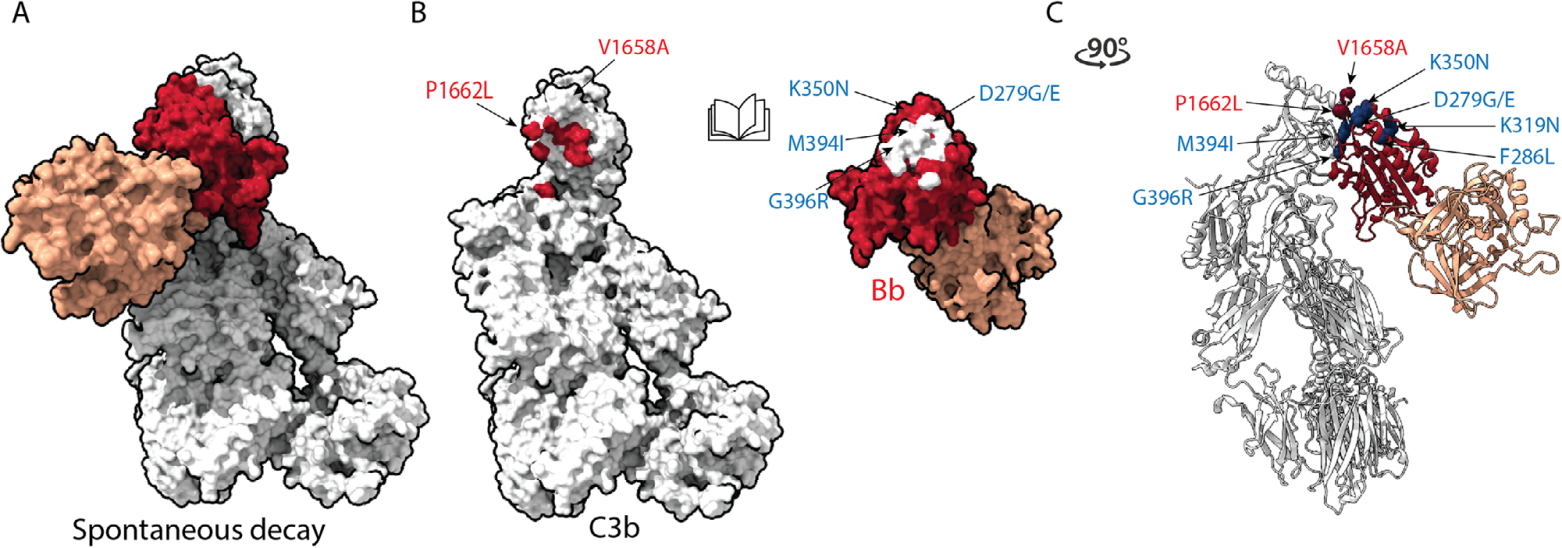
Mutants informing of C3bBb stability and spontaneous decay. (A) Surface representation of C3bBb (from PDB 9U62). (B) Open book representation of (A), highlighting the footprint of the interaction between the C345c domain of C3b and the vWA domain of Bb. Mutants impacting this interaction are indicated. Some variants informing of this interaction are not indicated because they are buried or hidden in the perspective represented. (C) 90° anticlockwise‐rotated ribbon representation of (A). Mutants in the C345c domain of C3b and the vWA domain of Bb impacting their interaction are represented as deep dark red (#67001f) and deep dark blue (#053061) spheres, respectively. C3b is represented in white (#f7f7f7), and Bb is domain‐colored (vWA is dark red #b2182b; SP is orange #f4a582).

C3bBb is a labile complex that dissociates spontaneously. Its dissociation is determined by the interactions between C3b and Bb, which are limited to the C345c domain of C3b and the vWA domain of Bb (Figures 5A,B and 2A,D). Interestingly, the few variants that alter the stability of C3bBb are located within this interaction footprint: those of FB are situated around MIDAS (S276, S280, and T353) [23, 24, 25], and those in C3b are located near the carboxyl‐terminal residue C345c (N1663), which binds to magnesium mediating the interaction (Table 4; Figure 5B,C) [26]. It is important to note that these variants in C3b and FB not only modify the stability of C3bBb but also the initial binding between C3b and FB, since the vWA surface comes into contact with the C345c domain of C3b throughout the C3bBb model (Figure 2B,E).

**TABLE 4:** Variants informing of C3bBb stability and spontaneous decay (*n* = 9).

Gene	cDNA	Exon	Protein	Domain	Informs about	Reference
**C3**	c.4973T>C	41	V1658A	C345c	Binding to vWA	[26]
	c.4985C>T	41	P1662L	C345c	Binding to vWA	[46]
**FB**	c.836A>G	6	D279G	vWA	Binding to C345c	[19, 40]
	c.837C>G	6	D279E	vWA	Binding to C345c	[19]
	c.858C>G	6	F286L	vWA	Binding to C345c	[19, 24]
	c.957A>T	7	K319N	vWA	Binding to C345c	[19]
	c.1050G>C	8	K350N	vWA	Binding to C345c	[19, 25]
	c.1182G>A	9	M394I	vWA	Binding to C345c	[19]
	c.1186G>A	9	G396R	vWA	Binding to C345c	[19]

**FIGURE 6:**
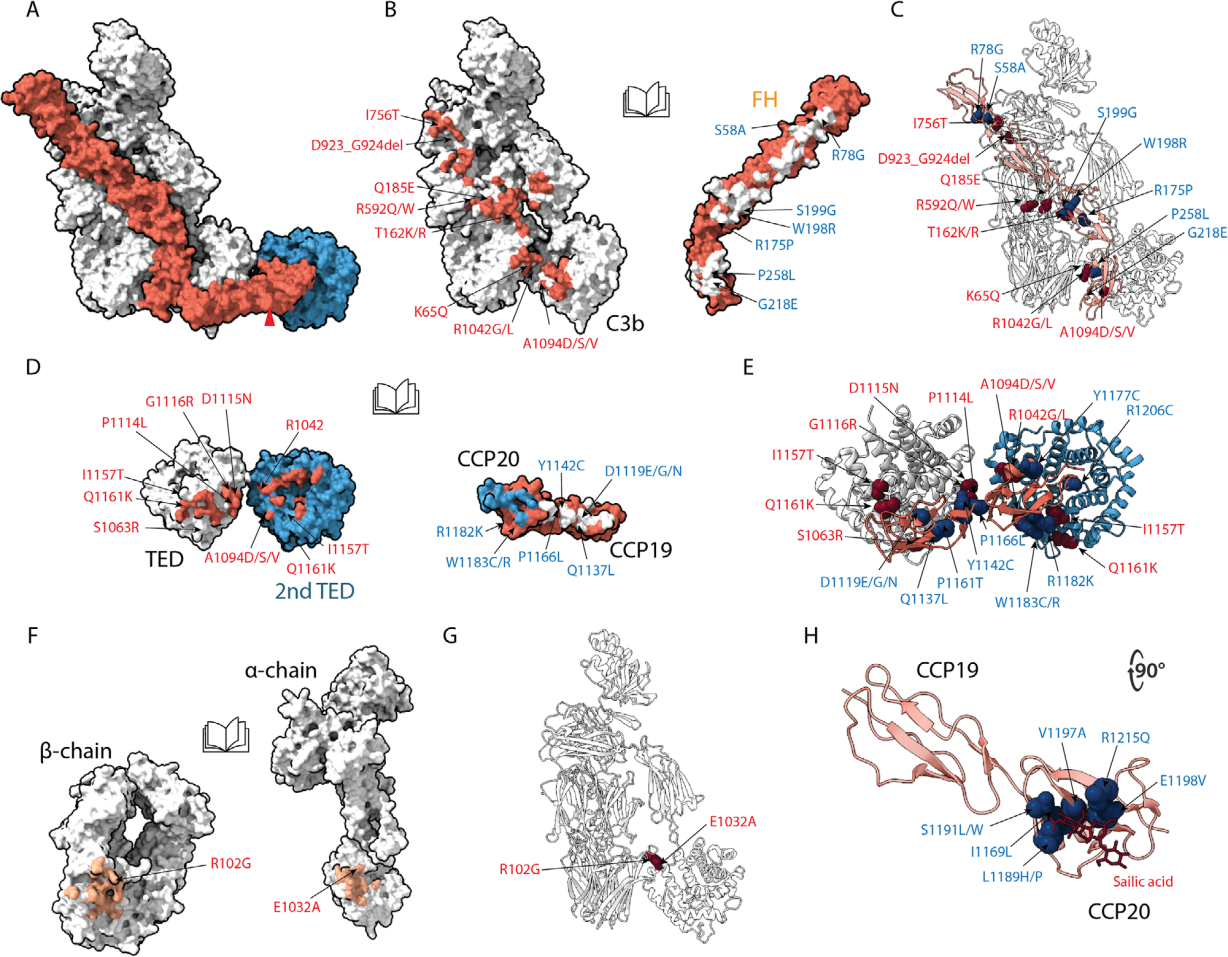
Mutants informing of FH binding to C3b for the DAA. (A) Surface representation of FH amino‐ (SCRs1‐4) and carboxyl‐terminal (SCRs19‐20) domains interacting with C3b (PDB 5O35) and a second TED domain (PDB 2XQW). Sialic acid position is indicated with a red triangle (PDB 4ONT). (B) Open book representation of (A), limited to the interaction between C3b and FH amino‐terminal domain. The footprint of their interaction is highlighted. Mutants impacting this interaction are indicated in red for C3b and blue for FH. (C) Ribbon representation of (A), limited to the interaction between C3b and FH amino‐terminal domain. Mutants in C3b and FH impacting their interaction are represented as deep dark red (#67001f) and deep dark blue (#053061) spheres, respectively. (D) Open book representation of (A), limited to the interaction between the FH carboxyl‐terminal domain and the 2 TED domains (PDB 2XQW). The footprint of their interaction is highlighted. Mutants impacting this interaction are indicated in red for C3b and blue for FH. (E) Ribbon representation of (A), limited to the interaction between the FH carboxyl‐terminal domain and the 2 TED domains (PDB 2XQW). Mutants in TED domains and FH impacting this interaction are represented as deep dark red (#67001f) and deep dark blue (#053061) spheres, respectively. (F) Open book representation of C3b (PDB 5O35), highlighting the footprint between TED (alpha chain) and MG1 (beta chain). Mutants informing about this interaction are indicated. (G) Ribbon structure of C3b (PDB 5O35). Mutants informing about MG1‐TED interaction are indicated as deep dark red (#67001f) spheres. (H) 90° upward ribbon representation of (A), focused on the interaction between the FH carboxyl‐terminal domain and sialic acids. Mutants in FH informing of this interaction are represented as deep dark blue (#053061) spheres. C3b is represented in white (#f7f7f7), the second TED domain is represented in blue (#4393c3), FH is represented in dark orange (#d6604d), and sialic acid is represented in dark red (#b2182b).

The decay of C3bBb can also be accelerated by RCA binding to the convertase to prevent uncontrolled complement amplification on host surfaces. With respect to this interaction, only the RCA binding to the C3b component is known, and FH is the RCA for which most variants have been characterized, informing us of this interaction. FH binds to C3b through its amino‐terminal (SCRS1‐4) and carboxy‐terminal (SCRs19‐20) domains (Figure 6A). Consistently, all variants in both C3b and FH that alter their interaction are located within, or near, their footprint (Table 5) (Figure 6B–E) [21, 27, 28, 29, 30, 31, 32, 33].

**TABLE 5:** Variants informing of FH binding to C3b and sialic acids for the performing DAA (*n* = 48).

Gene	cDNA	Exon	Protein	Domain	Informs about	Reference
**C3**	c.193A>C	2	K65Q	2	Binding to FH amino‐terminal	[27, 33]
	c.304C>G	3	R102G	3	Interaction MG1‐TED	[35]
	c.485C>G	4	T162R	4	Binding to FH amino‐terminal	[27]
	c.485C>A	4	T162K	4	Binding to FH amino‐terminal	[27]
	c.553C>G	5	Q185E	5	Binding to FH amino‐terminal	[27]
	c.1774C>T	14	R592W	14	Binding to FH amino‐terminal	[27, 29]
	c.1775G>A	14	R592Q	14	Binding to FH amino‐terminal	[27, 29]
	c.2267T>C	18	I756T	18	Binding to FH amino‐terminal	[28]
	c.2767_2774delACGGTG	22	D923_G924del	MG7	Binding to FH amino‐terminal	[21]
	c.3095A>C	24	E1032A	TED	Interaction MG1‐TED	[36]
	c.3124C>G	24	R1042G	TED	Binding to FH SCR4 and SCR20	[27]
	c.3125G>T	24	R1042L	TED	Binding to FH SCR4 and SCR20	[27]
	c.3189C>G; c.3187A>C	25	S1063R	TED	Binding to FH carboxyl‐terminal	[27]
	c.3280G>T	26	A1094S	TED	Binding to FH SCR4 and SCR20	[27]
	c.3281C>A	26	A1094D	TED	Binding to FH SCR4 and SCR20	[27]
	c.3281C>T	26	A1094V	TED	Binding to FH SCR4 and SCR20	[29]
	c.3341C>T	26	P1114L	TED	Binding to FH carboxyl‐terminal	[27]
	c.3343G>A	26	D1115N	TED	Binding to FH carboxyl‐terminal	[27, 29]
	c.3346G>A	26	G1116R	TED	Binding to FH carboxyl‐terminal	[27]
	c.3470T>C	27	I1157T	TED	Binding to FH carboxyl‐terminal	[27]
	c.3481C>A	27	Q1161K	TED	Binding to FH carboxyl‐terminal	[27, 29]
**FH**	c.172T>G	2	S58A	SCR1	Binding to C3b (MG‐ring, CUB and TED)	[31, 32]
	c.232A>G	2	R78G	SCR1	Binding to C3b (MG‐ring, CUB and TED)	[31, 32]
	c.524G>C	5	R175P	SCR3	Binding to C3b (MG‐ring, CUB and TED)	[31, 32]
	c.592T>C	5	W198R	SCR3	Binding to C3b (MG‐ring, CUB and TED)	[31, 32]
	c.595A>G	5	S199G	SCR3	Binding to C3b (MG‐ring, CUB and TED)	[31, 32]
	c.653G>A	6	G218E	SCR4	Binding to C3b (MG‐ring, CUB and TED)	[31, 32]
	c.773C>T	6	P258L	SCR4	Binding to C3b (MG‐ring, CUB and TED)	[31, 32]
	c.3355G>A	21	D1119N	SCR19	Binding to C3b (TED)—Surface 1	[31, 32]
	c.3356A>G	21	D1119G	SCR19	Binding to C3b (TED)—Surface 1	[31, 32]
	c.3357C>A/G	21	D1119E	SCR19	Binding to C3b (TED)—Surface 1	[31, 32]
	c.3410A>T	21	Q1137L	SCR19	Binding to C3b (TED)—Surface 1	[31, 32]
	c.3425A>G	21	Y1142C	SCR19	Binding to C3b (TED)—Surface 1	[31, 32]
	c.3481C>A	21	P1161T	SCR19	Binding to C3b (TED)—Surface 1	[31, 32]
	c.3497C>T	21	P1166L	SCR19	Binding to C3b (TED)—Surface 1	[31, 32]
	c.3505A>C	22	I1169L	SCR20	Binding to sialic acids	[31, 32]
	c.3530A>G	22	Y1177C	SCR20	Binding to C3b (TED)—Surface 2	[31, 32]
	c.3545G>A	22	R1182K	SCR20	Binding to C3b (TED)—Surface 2	[31, 32]
	c.3547T>A	22	W1183R	SCR20	Binding to C3b (TED)—Surface 2	[31, 32]
	c.3549G>T	22	W1183C	SCR20	Binding to C3b (TED)—Surface 2	[31, 32]
	c.3566T>A	22	L1189H	SCR20	Binding to sialic acids	[31, 32]
	c.3566T>C	22	L1189P	SCR20	Binding to sialic acids	[31, 32]
	c.3572C>T	22	S1191L	SCR20	Binding to sialic acids	[31, 32]
	c.3572C>G	22	S1191W	SCR20	Binding to sialic acids	[31, 32]
	c.3590T>C	22	V1197A	SCR20	Binding to sialic acids	[31, 32]
	c.3593A>T	22	E1198V	SCR20	Binding to sialic acids	[31, 32]
	c.3616C>T	22	R1206C	SCR20	Binding to C3b (TED)—Surface 2	[31, 32]
	c.3644G>A	22	R1215Q	SCR20	Binding to sialic acids	[31, 32]

More specifically, the amino‐terminal end of FH binds to the MG‐ring of C3b (MG1, MG2, MG6, and MG7), the CUB domain, and the TED. It is worth noting that although all SCRs at the amino‐terminus of FH leave a footprint on C3b, no variants have been found in SCR2 that affect its binding, and, curiously, there are also no variants in C3b that localize to the footprint of FH's SCR2 (Figure 6B,C). Regarding the binding of the carboxyl terminus of FH to C3b, two distinct surfaces that bind to the TED of C3b have been described by crystal structures and kinetic determinations: one in SCR19 and the other in SCR20 [34]. Both surfaces are extensively supported by disease‐associated variants in both FH and C3b (Figure 6D,E) [27, 30, 31, 32].

Some of the C3 variants on its TED (I1157T and Q1161K) are close enough to both FH interaction surfaces, making it uncertain whether they are affecting the interaction with FH SCR19 or SCR20 (Figure 6D,E). Similarly, both the amino‐ and carboxy‐terminal domains of FH bind to the C3b's TED with overlapping footprints. Although some variants in the TED (R1042G/L, A1094D/S/V, I1157T, and Q1161K) impact the binding of the FH carboxyl terminus [27], the overlapping footprints and the lack of specific assays make it unclear whether FH amino‐terminal binding is also affected (Figure 6B–E). Supporting their participation in FH amino‐terminal binding, the conformation of C3b, with MG1 in contact with the TED, has been proven to be important for FH binding. Spatially separating MG1 and TED splits the interaction surface by half and disrupts FH binding. Two variants (R102G and E1032), located within this footprint, impair the MG1‐TED interaction and therefore indirectly impair FH binding and regulation (see Figure 6F,G) [35, 36].

Bifunctionally, the carboxyl terminus of FH binds not only to C3b but also to sialic acids and other glycosaminoglycans, which is essential for the regulation of the C3bBb complex on host surfaces. Notably, several variants in the FH SCRs19‐20 impair its overall regulatory capacity without affecting binding to C3b, suggesting an impact on the recognition of other ligands [31, 32]. These variants are clustered in the sialic acid binding pocket (Figure 6H), and although the impact of these mutations on sialic acid binding was not formally determined in FH, additional studies did so for the equivalent residues to S1191 and V1197 in factor H‐related protein 1 (FHR‐1), C‐terminal domain of which is ∼98% identical to FH's SCRs19‐20, verifying the importance of sialic acid binding in FH‐mediated complement regulation on host surfaces [31, 32, 37].

**FIGURE 7:**
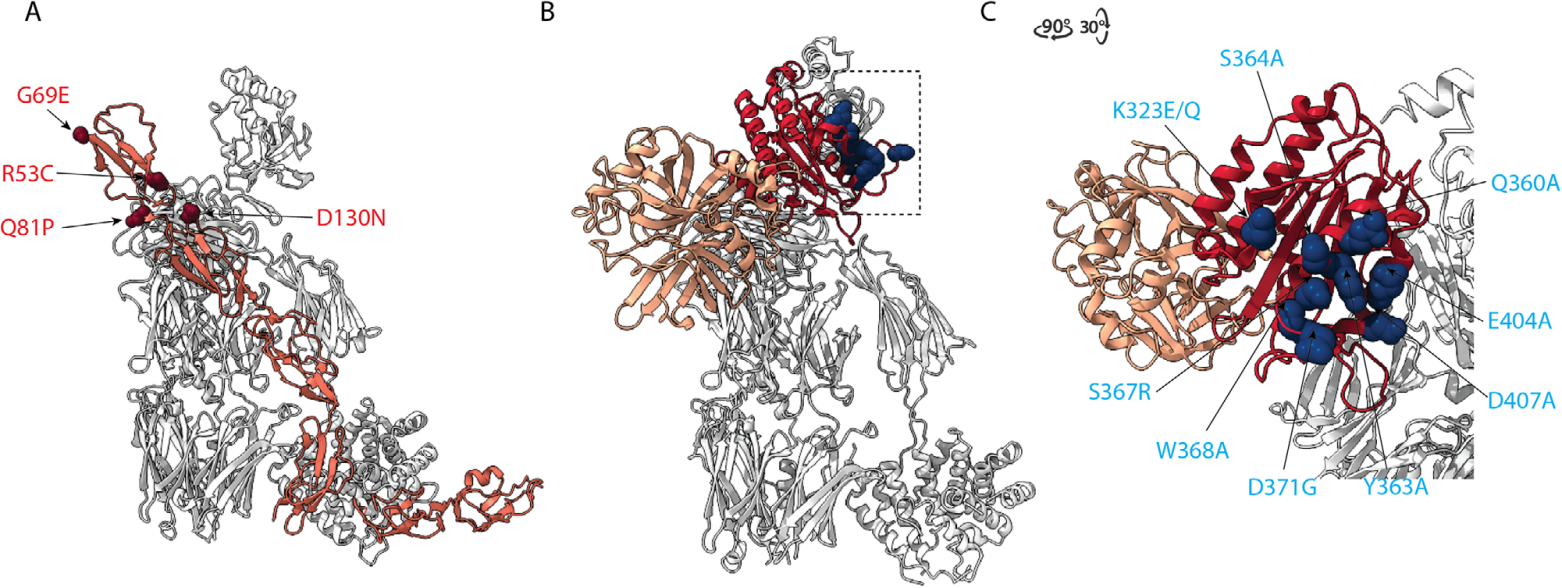
Variants informing about FH‐mediated DAA of C3bBb. (A) Ribbon representation of FH amino‐ (SCRs1‐4) and carboxyl‐terminal (SCRs19‐20) domains interaction with C3b (PDB 5O35). Mutants impacting the FH's DAA activity exclusively are represented as deep dark red (#67001f) spheres. (B) Ribbon representation of C3bBb (from PDB 9U62). Variants in FB impacting its resistance to FH‐mediated DAA activity exclusively are represented as deep dark blue (#053061) spheres. Dashed‐lined squares region is zoomed in panel C. (C) 90° clockwise and 30° downward rotated zoomed ribbon representation of (B). Variants in FB impacting its resistance to FH‐mediated DAA activity exclusively are represented as deep dark blue (#053061) spheres. C3b is represented in white (#f7f7f7), Bb is domain‐colored (vWA is dark red #b2182b; SP is orange #f4a582), and FH is represented in dark orange (#d6604d).

Apart from binding to C3b, little else is known about how RCAs accelerate the dissociation of C3bBb. Harris et al. [38] provided a clue by demonstrating that DAF binds to both C3b and Bb, and that binding to both is necessary to carry out DAA. Although this has not been formally confirmed for other RCAs with DAA, it is assumed to be true based on structural and functional homology. Accordingly, at least four variants have been identified in the amino‐terminal domain of FH that specifically prevent its DAA without altering binding to C3b (Table 6) [31, 32]. Interestingly, these variants are located on the surface opposite the one by which FH binds to C3b (Figure 7A). Similarly, several variants in FB, both disease‐associated [23, 24, 39] and laboratory‐designed [40], have revealed a specific impact on the resistance of C3bBb to FH regulation (Table 6). All these variants cluster on the same surface, which, interestingly, is also the surface on FB opposite the one closest to the C3b MG‐ring (Figure 7B,C). Future studies will decipher how these two surfaces participate in DAA and whether they define the DAF (or other RCAs) and FB interaction surfaces.

**TABLE 6:** Variants informing about FH‐mediated DAA of C3bBb (*n* = 14).

Gene	cDNA	Exon	Protein	Domain	Informs about	Reference
**FH**	c.157C>T	2	R53C	SCR1	FH's Surface relevant for DAA	[31, 32]
	c.206G>A	2	G69E	SCR1	FH's Surface relevant for DAA	[47]
	c.242A>C	2	Q81P	SCR1	FH's Surface relevant for DAA	[31, 32]
	c.388G>A	4	D130N	SCR2	FH's Surface relevant for DAA	[47]
**FB**	c.967A>G	7	K323E	vWA	Bb's Surface relevant for DAA	[23]
	c.967A>C	7	K323Q	vWA	Bb's Surface relevant for DAA	[23]
	*In‐lab designed*	8	Q360A	vWA	Bb's Surface relevant for DAA	[48]
	*In‐lab designed*	8	Y363A	vWA	Bb's Surface relevant for DAA	[48]
	*In‐lab designed*	8	S364A	vWA	Bb's Surface relevant for DAA	[48]
	c.1101C>A	8	S367R	vWA	Bb's Surface relevant for DAA	[39]
	*In‐lab designed*	8	W368A	vWA	Bb's Surface relevant for DAA	[48]
	c.1112A>G	8	D371G	vWA	Bb's Surface relevant for DAA	[19]
	*In‐lab designed*	9	E404A	vWA	Bb's Surface relevant for DAA	[48]
	*In‐lab designed*	9	D407A	vWA	Bb's Surface relevant for DAA	[48]

In regard to variants influencing DAA‐resistance of the C3bBb, it is worth noting that C3b and FB variants that increase the natural stability of the convertase also reduce FH‐mediated DAA. However, they do so via a different mechanism: while variants that increase C3bBb stability indirectly reduce FH‐mediated DAA due to a “stronger” binding between FB to C3b [24], those that exclusively alter DAA appear to affect the unknown mechanism of decay itself.

### 3.5 Inactivation of C3b to iC3b by FI

**FIGURE 8:**
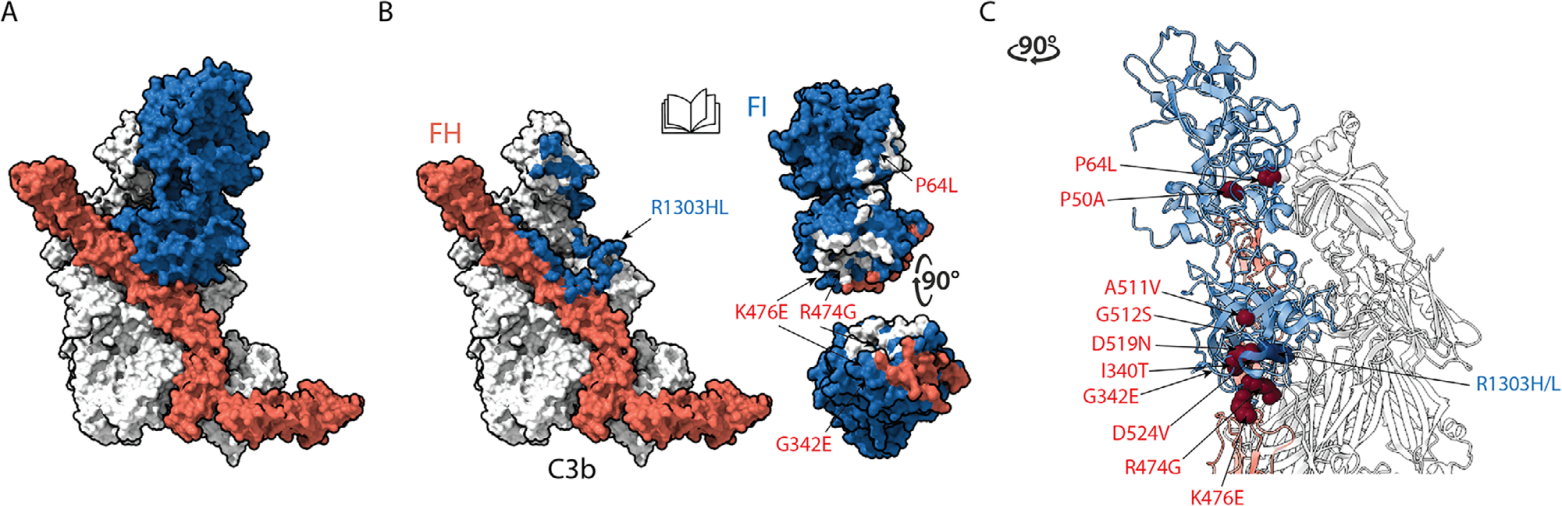
Mutants informing of C3b inactivation to iC3b by FI. (A) Surface representation of FI bound to C3b with FH as cofactor (PDB 5O32). (B) Open book representation of (A), highlighting the footprint between FI and C3b‐FH. Mutants impacting this interaction are indicated in red for FI and blue for C3b. (C) Zoomed 90° anticlockwise‐rotated ribbon representation of (A), focused on the interaction between FI and C3b. Mutants in FI and C3b impacting this interaction are represented as deep dark red (#67001f) and deep dark blue (#2166ac) spheres, respectively. C3b is represented in white (#f7f7f7), FH is represented in dark orange (#d6604d), and FI is represented in dark blue (#053061).

The C3bBb model is disrupted when C3b is inactivated to iC3b by FI and can no longer form a C3bBb. In this regard, the binding of the amino‐terminal end of FH to C3b is not only important for carrying out DAA but is also necessary to act as a cofactor in the inactivation of C3b, as it provides an additional binding surface for FI (Figure 8A) [41]. Unfortunately, no variants in FH have been found to specifically alter FI cofactor activity (FI‐CoA), but only as an indirect consequence of the impact on binding to C3b [30, 31, 32]. As for FI, a small number with functional impairment have been characterized (Table 7). FI interacts with the C345c and CUB domains of C3b through its FIM and SP domains, respectively (Figure 8A,B) [42, 43]. Accordingly, FI variants that affect its ability to inactivate C3b are located in these two domains. Those in the FIM domain are located within the interaction footprint, while most of those in the SP domain are partially buried (Figure 8B), delineating the “pocket” that binds to the cleavable loop (S1302‐R1303) in the CUB domain (Figure 8C). The only variants in C3b that provide information on FI inactivation are specifically located in the scissile loop and, although they are not functionally characterized, they modify the FI target sequence in C3b, which is a well‐known mechanism for disrupting enzymatic activity (Figure 8C).

**TABLE 7:** Variants informing of C3b inactivation to iC3b by FI (*n* = 12).

Gene	cDNA	Exon	Protein	Domain	Informs about	Reference
**C3**	c.3908G>A	30	R1303H	C345c	FI target	[49]
	c.3908G>T	30	R1303L	C345c	FI target	
**FI**	c.148C>G	2	P50A	FIM	Binding to C345c	[50]
	c.191C>T	2	P64L	FIM	Binding to C345c	[50]
	c.1019T>C	9	I340T	SP	Binding to CUB	[42, 43]
	c.1025G>A	9	G342E	SP	Binding to CUB	[42]
	c.1420C>G	11	R474G	SP	Binding to CUB	[42]
	c.1426A>G	11	K476E	SP	Binding to CUB	[50]
	c.1532C>T	12	A511V	SP	Binding to CUB	[42]
	c.1534G>A	12	G512S	SP	Binding to CUB	[42]
	c.1555G>A	13	D519N	SP	Binding to CUB	[42, 43]
	c.1571A>T	13	D524V	SP	Binding to CUB	[42, 43]

## 4 Conclusions

Functional characterization of mutants has proved to be a key tool in understanding the complement system. In this review, we contextualize more than 100 functionally significant variants informing about the current model of the formation, activity, and regulation of alternative pathway C3‐convertase, but also shedding light on undefined processes, such as the accelerated dissociation of C3 convertase mediated by RCAs. Future variant characterization will likely be helpful in elucidating other unknown processes, such as the mechanism behind the different performance between RCAs with similar activity (FH, DAF, and CR1 for DAA; FH, MCP, and CR1 for FI cofactor activity); the molecular composition of the C5‐convertase; and the interaction between C3b and C5 in the priming process [44].

The knowledge of variants condensed in this review also has translational applications. Variants contained within this review cluster in key functional spots that can provide clinicians and researchers with a “map” for interpreting novel variants while also delineating potential targets for anticomplement therapies that can modulate critical junctures in the complement cascade.

## Author Contributions

Héctor Martín Merinero, Seth J. Welsh, Yuzhou Zhang, and Richard J. H. Smith designed the review approach and collected the information. Zhen Xu generated all figures. Héctor Martín Merinero wrote the main draft. All authors reviewed the draft and agreed with the final version of the manuscript.

## Conflicts of Interest

Richard J. H. Smith serves as Director of the Molecular Otolaryngology and Renal Research Laboratories and is on the advisory board for Novartis. The remaining authors declare no conflicts of interest.
